# Slow‐channel myasthenia due to novel mutation in M2 domain of AChR delta subunit

**DOI:** 10.1002/acn3.50902

**Published:** 2019-09-27

**Authors:** Xin‐Ming Shen, Margherita Milone, Hang‐Long Wang, Brenda Banwell, Duygu Selcen, Steven M. Sine, Andrew G. Engel

**Affiliations:** ^1^ Department of Neurology and Neuromuscular Research Laboratory Mayo Clinic Rochester Minnesota; ^2^ Department of Neurology and Vesicular Biology Laboratory Mayo Clinic Rochester Minnesota; ^3^ Division of Neurology Department of Pediatrics Children's Hospital of Philadelphia Philadelphia Pennsylvania; ^4^ Department of Physiology and Biomedical Engineering and Receptor Biology Laboratory Mayo Clinic Rochester Minnesota; ^5^ Department of Pharmacology and Experimental Therapeutics Mayo Clinic Rochester Minnesota; ^6^ Department of Neurology Mayo Clinic Rochester Minnesota

## Abstract

**Objective:**

To characterize the molecular and phenotypic basis of a severe slow‐channel congenital myasthenic syndrome (SCCMS).

**Methods:**

Intracellular and single‐channel recordings from patient endplates; alpha‐bungarotoxin binding studies; direct sequencing of AChR genes; microsatellite analysis; kinetic analysis of AChR activation; homology modeling of adult human AChR structure.

**Results:**

Among 24 variants reported to cause SCCMS only two appear in the AChR δ‐subunit. We here report a 16‐year‐old patient harboring a novel δL273F mutation (δL294F in HGVS nomenclature) in the second transmembrane domain (M2) of the AChR δ subunit. Kinetic analyses with ACh and the weak agonist choline indicate that δL273F prolongs the channel opening bursts 9.4‐fold due to a 75‐fold increase in channel gating efficiency, whereas a previously identified εL269F mutation (εL289F in HGVS nomenclature) at an equivalent location in the AChR ε‐subunit prolongs channel opening bursts 4.4‐fold due to a 30‐fold increase in gating efficiency. Structural modeling of AChR predicts that inter‐helical hydrophobic interactions between the mutant residue in the δ and ε subunit and nearby M2 domain residues in neighboring α subunits contribute to structural stability of the open relative to the closed channel states.

**Interpretation:**

The greater increase in gating efficiency by δL273F than by εL269F explains why δL273F has more severe clinical effects. Both δL273F and εL269F impair channel gating by disrupting hydrophobic interactions with neighboring α‐subunits. Differences in the extent of impairment of channel gating in δ and ε mutant receptors suggest unequal contributions of ε/α and δ/α subunit pairs to gating efficiency.

## Introduction

The congenital myasthenic syndromes (CMS) are heterogeneous disorders associated with fatigable muscle weakness due to a compromised safety margin of neuromuscular transmission. Presently, no fewer than 33 CMS disease genes have been identified.^1^, ^2^, ^3^ The identified variants affect the development, stability, or signal transmitting capability of the neuromuscular junction. Approximately one‐half of the identified CMS are caused by mutations in different subunits of the acetylcholine receptor (AChR).

The AChR is a pentameric ligand‐gated cation ion channel composed of homologous subunits with stoichiometry (α1)_2_β1δε. Each receptor has two binding sites; a high‐affinity site formed between α‐ and δ‐subunits, and a low affinity site between α‐ and ε‐subunits. The α‐subunit forms the principal face of each binding site, and the δ‐ and ε‐subunits form the complementally face. Each subunit contains four transmembrane domains, with the second transmembrane domain (M2) of each subunit forming the wall of the ion channel. Within M2, hydrophobic residues conserved among subunits contribute to channel gating, but whether residues at equivalent positions of the subunits contribute equivalently to channel gating remains unclear.

Mutations of AChR subunits can reduce expression of the receptor on the cell surface, alter the kinetics of receptor activation, or both. CMS with kinetic defects are divided into two categories, fast‐channel CMS (FCCMS) and slow‐channel CMS (SCCMS). The SCCMS exhibit slow decay of miniature endplate potentials (MEPPs) or currents (MEPCs) either due to prolonged channel opening or increased channel reopening.^4^ During physical activity, prolonged EPPs sum in a staircase manner, which depolarizes the postsynaptic membrane and causes depolarization block that inactivates voltage‐gated sodium channels. In addition, prolonged EPPs allow excessive calcium influx into the postsynaptic region, which initiates focal degeneration of the junctional folds, loss of AChR and apoptosis of subjunctional nuclei, collectively referred to as an endplate myopathy.^5^ Moreover, AChRs from SCCMS patients are prone to desensitization, which reduces the number of AChRs available for activation.^6^ To date, 24 slow‐channel mutations have been reported in different domains of AChR subunits,^7^ but only two mutations involving the same residue were identified in the δ subunit.^8^, ^9^ Most slow‐channel mutations appear in transmembrane domains of AChR subunits, but detailed analysis of their kinetic consequences has been hindered by the inability of the recording instruments to capture the fastest gating steps with ACh as agonist. Hence choline, a weak agonist eliciting a slower rate of channel opening than ACh, has been used to study kinetics of activation of SCCMS mutations.^7^, ^10^, ^11^

In this study, we report a patient harboring a novel L273F mutation in the M2 domain of the AChR δ subunit, evaluate its pathogenic effects by clinical and morphologic studies, and examine the kinetic consequences of the mutation with choline as agonist. We also compare the kinetic consequences of δL273F to those of a previously reported εL269F mutation^12^ located at a position equivalent to that of δL273F.

## Patients and Methods

### Participants and ethical approval

This study was approved by the Mayo Clinic Institutional Review Board. The patient’s parents gave informed consent for the patient to participate in the study.

### Structural studies of endplates

Morphologic parameters of endplates (EP) in patient’s intercostal muscle were determined as previously described.^13^, ^14^, ^15^, ^16^, ^17^ Details of the procedure are described in Data S1.

### In vitro electrophysiology studies of endplates

The amplitude of the miniature EP potential (MEPP), the quantal content of the EP potential (*m*), estimates of the probability of quantal release (*p*), the number of readily releasable quanta (*n*), and the EP AChR content were determined as previously described.^18^, ^19^, ^20^, ^21^

### Mutation analysis

We directly sequenced genes encoding the AChR α‐, β‐, δ‐, and ε‐subunits using the patient’s genomic DNA as previously described.^4^ For paternity check, we analyzed four microsatellite markers (D11S1344, D11S4109, D11S4117, and D11S4174) using the GeneScan Analysis software (Applied Biosystems, Foster City, CA) as previously described.^11^

### Construction and expression of wild‐type and mutant AChR

Preparation of plasmids and expression of wild‐type and mutant AChR in HEK cells were as previously described.^22^ Details of the procedure are described in Data S1.

### α‐Bungarotoxin binding measurements of wild‐type and mutant AChR

The total number of ^125^I‐α‐bgt sites on the surface of transfected HEK cells expressing wild‐type and mutant AChR and estimates of overall dissociation constants were determined as previously described.^23^, ^24^ Details of the procedure are indicated in Data S1.

### Patch‐clamp recordings and single‐channel kinetic analysis of wild‐type and mutant AChR

Single‐channel patch‐clamp recordings of wild‐type and mutant receptors were performed as previously described.^4^, ^25^, ^26^, ^27^ Estimates of rate constants underlying AChR activation with partial agonist choline were determined as previously described.^7^, ^10^, ^11^, ^28^ Data S1 describes details of the procedure.

### Homology modeling of adult human muscle AChR

A homology model of the adult human muscle AChR was generated using version 9.0 of the program MODELER^29^ using spatial restraints provided by the following x‐ray crystal structures: mouse serotonin 5‐HT_3_ receptor (PDB# 4PIR),^30^ a pentameric alpha7 ligand binding domain chimera (PDB# 3SQ9),^31^ and C. elegans glutamate‐gated chloride channel (PDB# 3RHW).^32^ The refine 1 mode in MODELER, which uses conjugate gradients with simulated annealing and molecular dynamics, was used. Modeling included polar hydrogens but excluded nonpolar hydrogens.

## Results

### Clinical findings

**Figure 1:**
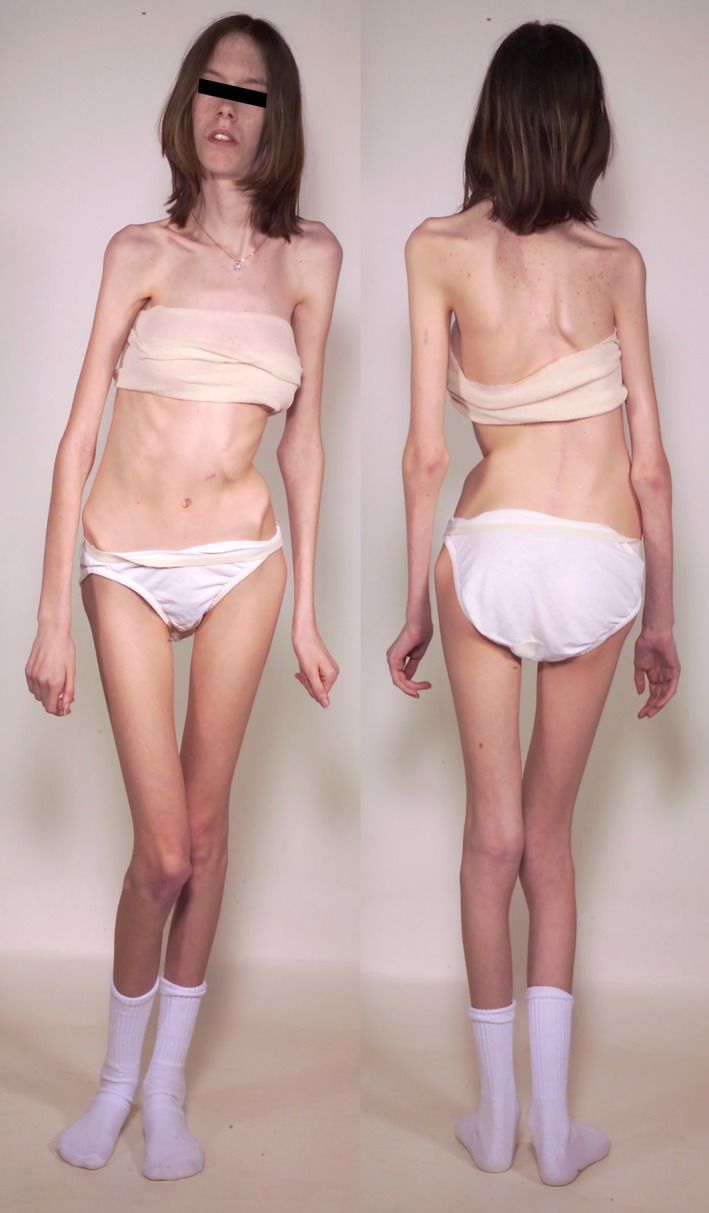
Patient at age 16. Note marked scoliosis, increased lordosis, and severe diffuse muscle atrophy.

A 16‐year‐old girl had gradually worsening myasthenic symptoms since infancy (Fig. 1). She sat up at 12 months and walked at 18 months but fell frequently. She had eyelid ptosis at age 2 years and restricted ocular ductions at age 5 years. Later she developed increasing scoliosis, and weakness and atrophy of cervical, limb and torso muscles, difficulty swallowing and became partially wheelchair‐dependent. EMG studies showed a repetitive compound muscle action potential and a 15% to 25% decrement of the fourth compared with the first evoked compound muscle action potential on 2 Hz stimulation in different muscles.

### Endplate studies

**Figure 2:**
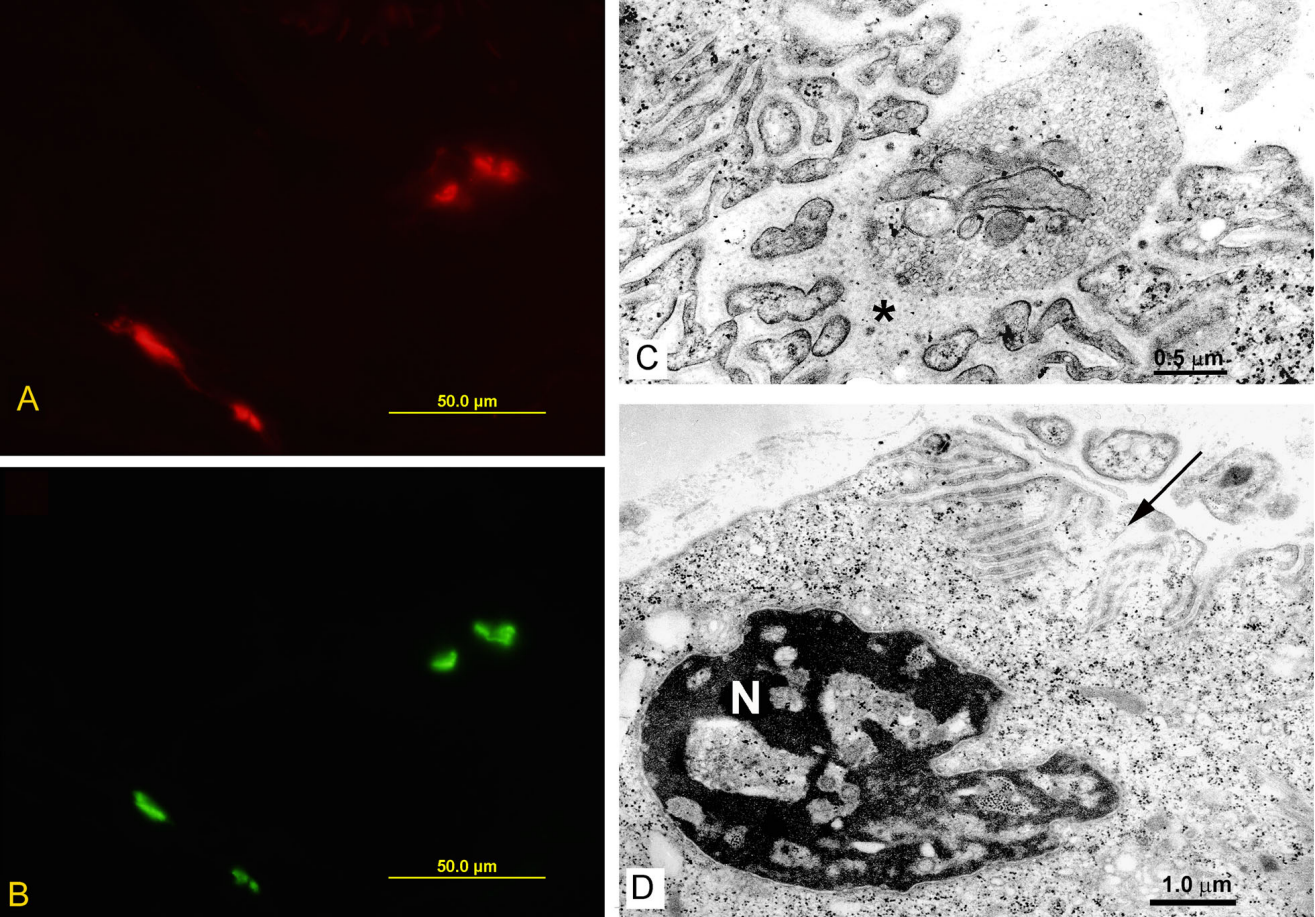
Immunofluorescence localization of AChR in red (A) and of AChE in green (B) at patient’s EP. EM studies of patient’s endplates (C) and (D). Note degenerating junctional folds and widened synaptic space (asterisk) (C). Note apoptotic nucleus (N) and abandoned postsynaptic region (arrow) (D).

**Figure 3:**
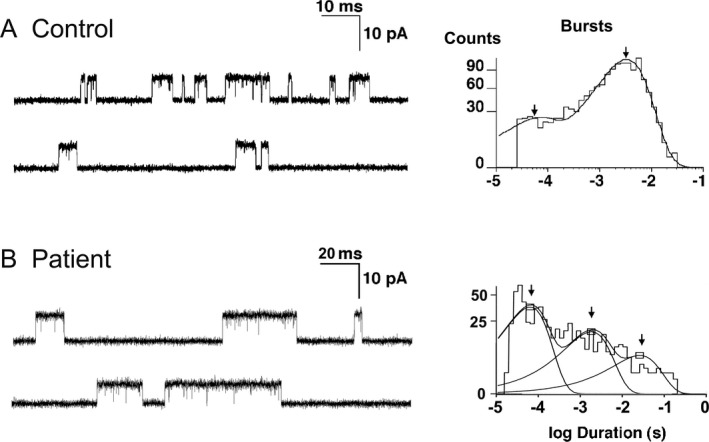
Single‐channel events recorded from control and patient EPs. Note prolonged opening bursts at patient EP. Bandwidth is 12 kHz at control and 11.7 kHz at patient. ACh concentration is 1 μmol·L^−1^ at control and 50 nmol·L^−1^ at patient.

The configuration of EPs, indicated by the cytochemical reaction for acetylcholinesterase on teased muscle fibers, was normal (Fig. 2A and B). Electron microscopy revealed degenerating junctional folds, abandoned postsynaptic regions, and apoptotic junctional nuclei (Fig. 2C and D). Table 1 summarizes in vitro recordings from EPs. The mean miniature endplate potential (MEPP) amplitude was in the high normal range. Both MEPPs and endplate potentials (EPPs) decayed bi‐exponentially with the second component 8.2 and 5.6 times longer than control, respectively. Quantal release by nerve impulse fell in the low normal range. Single‐channel patch‐clamp recordings from 6 EPs revealed an abnormal third component in the burst duration histogram that was 8.5‐fold longer than the longest component observed at control EPs (Fig. 3A and B, Table 1).

**Table 1:** Endplate studies.

	Patient	Controls
MEPP		
Amplitude (mV)^1^	1.30 ± 0.25 (6)	1.00 ± 0.025 (165)
Decay (msec)		
τ_1_	4.07 ± 0.12 (3)	3.75 ± 0.14 (46)
τ_2_	30.57 ± 5.22 (3)	
EPP^2^		
Decay (msec)		
τ_1_	2.39 ± 0.64 (13)	4.55 ± 0.11 (190)
τ_2_	25.69 ± 4.12 (13)	
Quantal content (1 Hz)^3^	23 ± 2 (13)	31 ± 1 (190)
Range	13–36	8.4–87
Single‐channel opening bursts		
τ_1,_ msec	0.10 ± 0.019 (6)	0.12 ± 0.012 (32)
a_1_	0.53 ± 0.038	0.16 ± 0.014
τ_2_, msec	2.08 ± 0.36 (6)	3.04 ± 0.18 (32)
a_2_	0.35 ± 0.36	0.85 ± 0.015
τ_3_, msec	25.87 ± 2.50 (6)	
a_3_	0.12 ± 0.012	

Values indicate mean ± SEM; Measurements at 29°C ± 0.5°C for MEPPs and EPPs recordings and 25°C ± 0.5°C for single‐channel recordings. Numbers in parenthesis indicate number of subjects for [^125^I]α‐bgt binding sites/EP and number of EPs for other measurements. τ_n_ and a_n_ indicate decay time constants and fractional histogram areas. ACh = 1 µmol·L^−1^ for control EPs and 50 nmol·L^−1^ for patient EPs.

^1^ Corrected for resting membrane potential of −80 mV and a fiber diameter of 50 μm.

^2^ Corrected for resting membrane potential of −80 mV.

^3^ Corrected for a resting membrane potential of −80 mV, nonlinear summation, and non‐Poisson release.

### Mutation analysis

**Figure 4:**
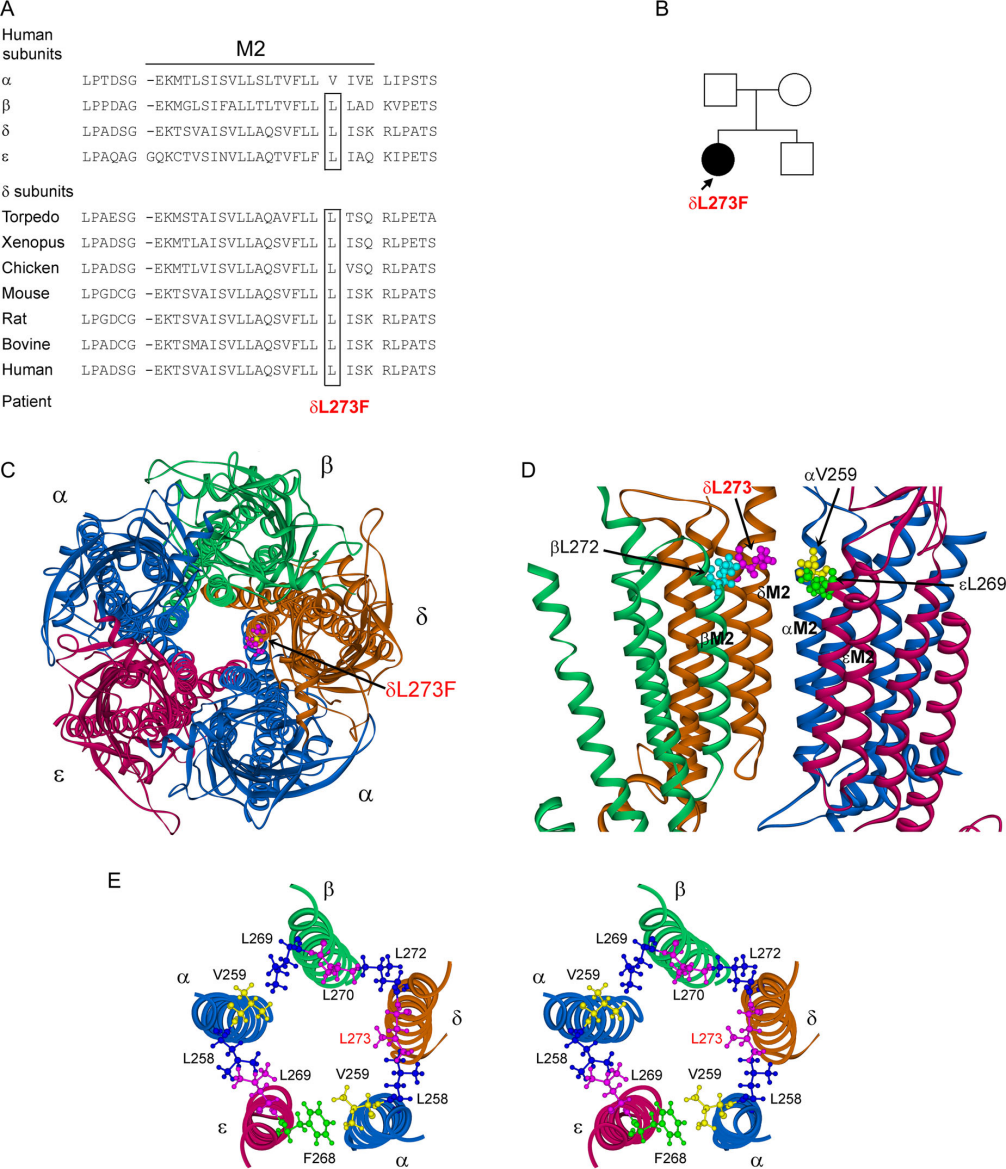
(A) Multiple alignment of the AChR M2 domain**.** Leucine at codon 273 is conserved across all non‐α human AChR subunits and δ subunits of different species. (B) δL273F is present in propositus but not in her parents and her brother. (C) Structural model of extracellular and transmembrane domains of human adult muscle AChR viewed from the synaptic space indicating position of mutant residue L273 (pink) in M2 domain of δ subunit. Model of homology human adult muscle AChR is based on the crystal structures of mouse serotonin 5‐HT_3_ receptor (4PIR), pentameric alpha7 ligand binding domain chimera (3SQ9) and C. elegans glutamate‐gated chloride channel (3RHW). (D) Side view of transmembrane domains indicating mutant residue δL273 and its equivalent residues in other subunits. One α subunit is omitted for clarity. (E) Stereo view of M2 domains of all subunits viewed from the synaptic space indicating 5 pairs of interactions between neighboring subunits. Mutant residue δL273 and its equivalent residues in other subunits (αV259, εL269, and βL270) interact with corresponding downstream residue of each in neighboring subunit, respectively. Five pairs of interaction residues between neighboring subunits are δL273/αL258, αV259/εF258, εL269/αL258, αV259/βL269, and βL270/δL272.

Direct sequencing of genes encoding AChR subunits revealed a heterozygous C‐to‐T mutation in *CHRND* exon 8 at position 233396121C>T (GRCh38.p12) on chromosome 2, g.5419C>T, or c.880C>T, that predicts a leucine‐to‐phenylalanine substitution at amino acid 294 of the peptide (p.δL294F) with the HGVS nomenclature, or at amino acid 273 of the mature peptide (p.δL273F) using the legacy nomenclature. Of note, in the HGVS nomenclature, the first nucleotide is the A of the ATG initiation codon, whereas in legacy nomenclature the first nucleotide is the first base of a cDNA segment encoding the mature peptide. As the AChR delta subunit carries a signal peptide of 21 amino acids, the first codon in legacy nomenclature is codon 22 in HGVS nomenclature. Causative mutations in AChR subunits related to congenital myasthenic syndrome are all reported with positions of mutant residues in the mature peptide. Amino acid numbers with legacy nomenclature correspond to those in the crystal structure of the protein. In this study, we employ the legacy nomenclature (p.δL273F). The indicated codon numbers start with the first codon of the mature peptide (NP_000742.1), and the indicated nucleotide numbers start from the translational start site, with +1 corresponding to the A of the ATG translation initiation codon (NM_000751.3). The mutated residue is located in the second transmembrane domain (M2) of the AChR δ‐subunit; it is conserved across non‐α‐subunits of human AChR and across δ‐subunits of different species (Fig. 4A). The mutation was not detected in the patient’s parents and brother (Fig. 4B). Analysis of four microsatellite markers (D11S1344, D11S4109, D11S4117, and D11S4174) in all family members showed 99.97% probability of paternity. Thus, δL273F is of germ‐line origin. The mutant residue δL273 faces the neighboring α subunit (Fig. 4C–E) and is positioned near the extracellular end of M2 (Fig 4D). δL273F was not found in available variant databases.

**Figure 5:**
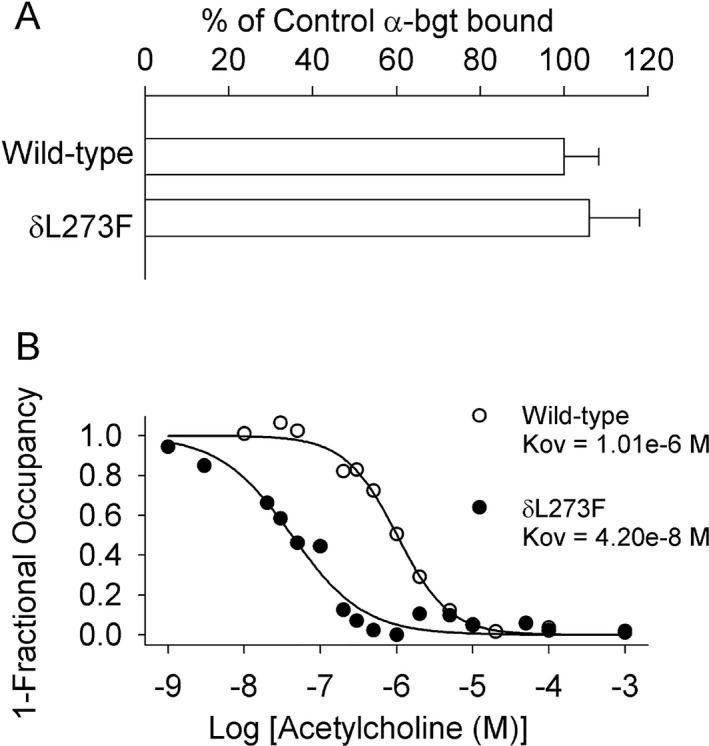
(A) Specific [^125^I]α‐bgt binding to surface receptors on intact HEK cells transfected with wild‐type subunits and with the indicated δ subunit and wild‐type α, β, and ε subunits. The results are normalized for α‐bgt binding to wild‐type AChR and represent the mean and SD of 6 and 3 experiments for wild‐type and mutant. (B) ACh binding to intact HEK cells transfected with indicated AChR subunits determined by competition against the initial rate of [^125^I]α‐bgt binding. Smooth curves are fits of data by Hill equation 1‐Y = 1/(1+([ACh]/K_ov_)^n^), Y: fractional occupancy by ACh; n: Hill coefficient (1.27 and 0.92 for wild‐type and mutant); K_OV:_ overall dissociation constant.

**Figure 6:**
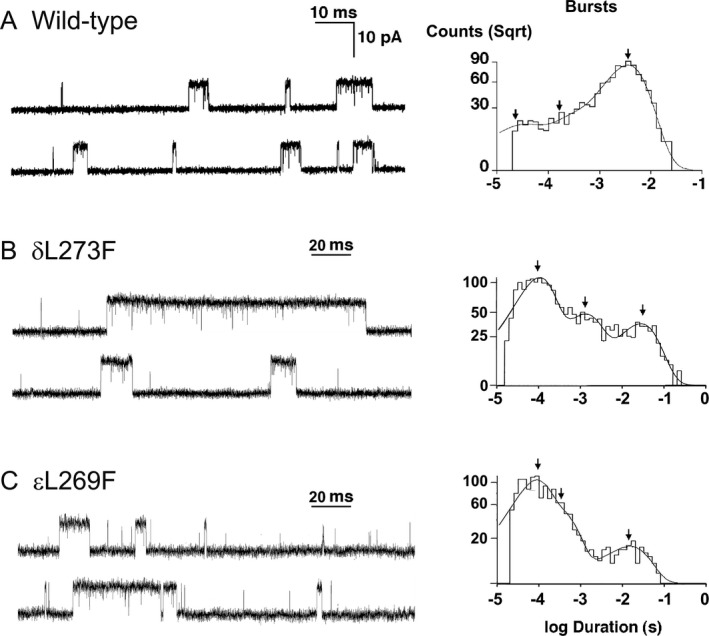
Single‐channel events recorded from HEK cells expressing wild‐type (A), δL273F (B) and εL269F‐ AChR (C). Note prolonged channel events at mutant AChRs. Bandwidth is 12 kHz in wild‐type AChR and 11.7 kHz at mutant AChRs. ACh concentration is 50 nmol·L^−1^ for both wild‐type and mutant AChRs.

**Figure 7:**
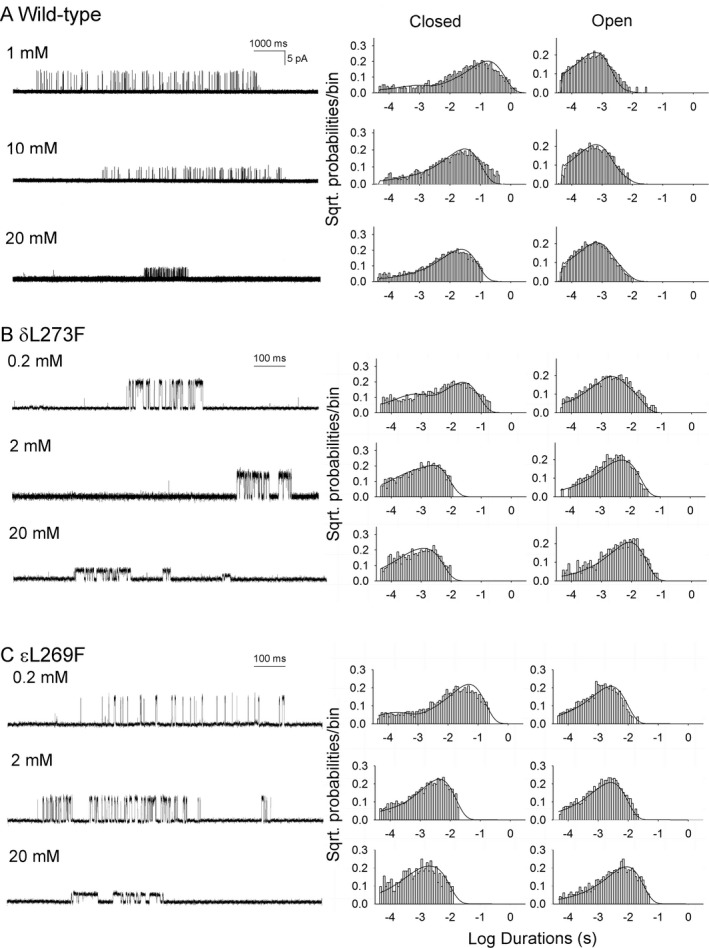
Kinetics of activation of wild‐type AChR (A), δL273F (B) and εL269F‐ AChR (C). Left column: Single clusters of channel openings recorded at indicated choline concentrations from HEK cells. Center and right columns: Corresponding closed and open duration histograms with superimposed probability density functions for the adopted scheme (Scheme 1 in text) of receptor activation for the entire range of choline concentrations (1, 2, 10, 20 mmol·L^−1^ in wild‐type, 0.2, 0.5, 1, 2, 5, 20 mmol·L^−1^ for mutants). Table 3 lists fitted rate constants.

### Surface expression of mutant receptors

We engineered the δL273F mutation into CHRND cDNA and co‐expressed it with complementary wild‐type AChR subunit cDNAs in HEK293 cells. [^125^I]α‐bungarotoxin binding measurements indicated normal expression of δL273F‐AChR on the cell surface (Fig. 5A). To compare the apparent agonist affinity of mutant and wild‐type AChRs, we measured ACh binding at steady state by competition against the initial rate of [^125^I]α‐bungarotoxin binding to intact cells. The apparent dissociation constant for the δL273F‐AChR was 24‐fold smaller than that of wild‐type AChR (Fig. 5B), suggesting the mutation promotes functional states with high affinity for the agonist, such as the open‐channel state, the desensitized state or both.

### Single‐Channel Recordings of δL273F AChR Expressed on HEK293 Cells

To evaluate the kinetic consequences of δL273F, we recorded single‐channel currents activated by a limiting low concentration of ACh (50 nmol·L^−1^) applied to HEK293 cells expressing wild‐type and mutant AChRs. For both wild‐type and δL273F‐AChRs, single‐channel currents appear as isolated single openings or as bursts of several openings in quick succession (Left columns in Fig. 6A and B). Histograms of single‐channel openings and burst durations exhibit three exponential components (Right columns in Fig. 6A and B, and Table 2) corresponding to two brief monoliganded and one long diliganded open states. Relative to the wild‐type AChR, δL273F increased the length of the longest component of open intervals and open bursts 5.1‐ and 9.4‐fold, respectively (Table 2). The changes of open intervals and burst durations are almost identical to those found at the patient’s end‐plates by single‐channel patch clamp recordings (Table 1), indicating that the δL273F mutation is the cause of the SCCMS.

**Table 2:** Open interval and burst durations of wild‐type and mutant AChRs in HEK cells.

Agonist		ACh			Choline	
AChR	Wild‐type	δL273F	εL269F	Wild‐type^2^	δL273F	εL269F
Patches	21	5	4	4	4	4
*Open Intervals*						
τ _1_, msec (a_1_)	0.040 ± 0.03^1^ (0.17 ± 0.02)	0.11 ± 0.011 (0.37 ± 0.037)	0.061 ± 0.0090 (0.43 ± 0.063)	0.069 (0.069)	0.045 ± 0.010 (0.24 ± 0.024)	0.021 ± 0.0043 (0.46 ± 0.080)
τ _2,_ msec (a2)	0.30 ± 0.05 (0.27 ± 0.04)	2.08 ± 0.24 (0.34 ± 0.055)	0.32 ± 0.033 (0.27 ± 0.062)	0.33 (0.55)	1.59 ± 0.040 (0.53 ± 0.053)	0.29 ± 0.073 (0.25 ± 0.047)
τ _3,_ msec (a3)	1.35 ± 0.05 (0.67 ± 0.04)	6.84 ± 0.65 (0.29 ± 0.049)	2.29 ± 0.054 (0.29 ± 0.023)	0.73 (0.38)	5.16 ± 0.25 (0.23 ± 0.072)	2.10 ± 0.21 (0.29 ± 0.061)
*Bursts*						
τ _1_, msec (a1)	0.04 ± 0.002^1^ (0.24 ± 0.02)	0.11 ± 0.015 (0.57 ± 0.024)	0.084 ± 0.015 (0.54 ± 0.0073)	0.069 (0.059)	0.050 ± 0.0079 (0.44 ± 0.088)	0.13 ± 0.061 (0.54 ± 0.080)
τ _2,_ msec (a_2_)	0.47 ± 0.06 (0.21 ± 0.03)	1.22 ± 0.13 (0.24 ± 0.015)	0.35 ± 0.046 (0.34 ± 0.019)	0.41 (0.64)	1.18 ± 0.10 (0.30 ± 0.054)	0.74 ± 0.25 (0.29 ± 0.093)
τ _3,_ msec (a_3_)	3.31 ± 0.12 (0.58 ± 0.04)	31.26 ± 3.81 (0.19 ± 0.021)	14.45 ± 0.49 (0.12 ± 0.021)	0.82 (0.31)	6.72 ± 0.82 (0.27 ± 0.075)	2.95 ± 0.39 (0.17 ± 0.041)

Values indicate means ± SE. Membrane potential −80 mV; bandwidth 10 – 12 kHz; temperature 25°C ± 0.5°C. τ_n_ and a_n_ indicate decay time constants and fractional histogram areas.

^1^ Not detected at 12 and 3 patches, respectively;

^2^ Analyzed from 4 patches combined due to infrequent openings. ACh 50 nmol·L^−1^; Choline 50 μmol·L^−1^ for wild‐type, 20 μmol·L^−1^ for mutants.

To further define the kinetic steps altered by δL273F, we recorded single‐channel currents activated by a range of choline concentrations. Choline is a weak agonist and has been used to quantify kinetic changes caused by gain‐of‐function AChR mutations, in part because it exhibits an inherently slow rate of channel opening.^10^ The δL273F receptors exhibited clusters of openings with 0.2 mmol·L^−1^ choline, whereas the wild‐type receptor failed to do so with choline concentrations less than 1 mmol·L^−1^, suggesting δL273F enhances desensitization.

Single‐channel currents activated by a saturating concentration of choline (20 mmol·L^−1^) appeared in clusters of closely spaced openings flanked by prolonged closed periods (Fig 7A–C). The reciprocal of the mean of intracluster closed intervals gives an estimate of the channel opening rate constant *β*.^10^, ^11^, ^28^ We fitted the following minimal kinetic scheme (Scheme 1) to open and closed dwell times obtained over a range of choline concentrations:

(Scheme 1) $$\begin{array}{c} A+R\underset{k_{-1}}{\overset{k_{+1}}{⟷}}\text{AR}+A\underset{k_{-2}}{\overset{k_{+2}}{⟷}}A_{2}R\\ \;\underset{\alpha }{\overset{\beta }{⟷}}A_{2}R^{*}\underset{K_{-b}}{\overset{K_{+b}}{⟷}}A_{2}R_{B} \end{array}$$

In this scheme, two agonists (A) bind to the receptor (R) with association rate constants k_+1_ and k_+2_ and dissociate with rate constants k_‐1_ and k_‐2_. The doubly occupied receptor opens with a rate constant *β* and closes with a rate constant α; Agonist blocks the open channel with a rate constant k_+b_, and unblocks from the open channel with a rate constant k_–b_. Owing to bandwidth limitations, the previously identified closed state between A_2_R and A_2_R*, known as flip or primed,^33^, ^34^ is not included in this scheme, thus the fitted rates β, α, and k_–2_ are apparent rate constants. Also, a desensitized state is not included because each cluster begins following recovery from desensitization and ends upon return to the desensitized state, so that closings within clusters represent transitions between activatable states. We determined the rate constants in the kinetic scheme under the simplifying assumption that the association and dissociation rate constants are equivalent at each binding site. The rate constant for channel opening is the reciprocal of the mean of intracluster closed intervals obtained at a saturating concentration (20 mmol·L^−1^) of choline. Using Scheme 1, global fitting of open and closed times over a range of choline concentrations showed that δL273F increases the gating equilibrium constant (θ), defined by β/α, 75‐fold, by speeding the channel opening rate constant (β) 13‐fold and slowing the closing rate constant (α) 5.7‐fold. (Fig. 7A and B and Table 3). The dissociation constant of choline for the closed state (K_d_) is enhanced by only 2.9‐fold (Table 3).

**Table 3:** Kinetic parameters of wild‐type and mutant AChRs expressed in HEK cells with choline as agonist.

Rate constants	Wild‐type	δL273F	εL269F
k_+1_ (mM^‐1^sec^‐1^)	698 ± 193	3227 ± 179	8080 ± 713
k_‐1_ (sec^‐1^)	539 ± 96	851 ± 83	2760 ± 418
β (sec^‐1^)	48 ± 1	625 ± 22	429 ± 54
α (sec^‐1^)	1284 ± 360	225 ± 3	385 ± 5
K_d_ (mM)	1.54	0.53	0.68
θ	0.037	2.78	1.11
Predicted burst length, msec	0.81	6.08	2.80
Low conc burst length, msec	0.82	6.72	2.95

Two agonist molecules (A) bind to the closed receptor (R) with association rate constants k_+1_ and k_+2_, and dissociate with rate constants k_‐1_ and k_‐2_ (Scheme 1). The association and dissociation rate constants were obtained by assuming the two binding sites were equivalent as follows: k_+1_ = 2 k_+2_, and k_‐2_ = 2 k_‐1_. The microscopic dissociation equilibrium constant of each site (K_d_) = k_‐1_/k_+2_. The gating equilibrium constant θ = opening rate (β)/closing rate (α). The predicted burst length was derived from (1 + β/ k_‐2_)/α. Bandwidth = 4 kHz.

To validate the fitted rate constants, we measured channel burst durations for wild‐type and mutant AChRs in the presence of limiting low concentrations of choline (20 μmol·L^−1^ for mutant, 50 μmol·L^−1^ for wild type) and compared these to burst durations predicted by the fitted rate constants. At low concentrations of choline, channel openings appear as bursts of several openings in quick succession, with each burst arising from a different channel. The predicted burst durations, given by (β/k_–2_ + 1)/α, are 0.81msec for the wild‐type AChR and 6.08 msec for the δL273F AChR, in good agreement with the measured burst durations (0.82 and 6.72 msec, respectively) (Tables 2 and 3). Thus the dominant effect of δL273F is to markedly enhance the efficiency of channel gating.

### Single‐Channel Recordings of εL269F AChR Expressed on HEK293 Cells

The εL269F mutation has been identified in six unrelated SCCMS patients,^9^, ^12^, ^35^, ^36^, ^37^, ^38^ and is equivalent to the δL273F mutation in both sequence alignments and three‐dimensional structure. However, the kinetic effects of εL269F were not analyzed in detail in previous studies. In single‐channel recordings from EPs of a patient carrying εL269F, we found that the open burst durations were prolonged 3.5‐ fold compared to controls^12^, whereas the δL273F mutation in the current patient prolonged the open burst duration 8.5‐fold (Table 1). To confirm the functional difference between the two patients, we also examined channel opening events in HEK cells expressing the εL269F‐AChR at a low concentration of ACh (50 nmol·L^−1^). With the εL269F mutation, the longest components of open intervals and opening bursts were 1.7 and 4.4‐fold greater than for wild‐type, whereas the corresponding values for δL269F were 5.1‐fold and 9.4‐fold greater than for wild‐type (Fig 6A–C; Table 2). To identify the elementary functional step that prolongs the opening events in the ε and δ subunits, we recorded channel openings elicited by 0.2 mmol·L^−1^ to 20 mmol·L^−1^ choline. As shown in Figure 7, choline generated openings from the εL269F‐AChR cluster more tightly than wild‐type but less tightly than the δL273F‐AChR (left columns in Fig. 7A–C). Fitting Scheme 1 to open and closed dwell times obtained over the full range of choline concentrations revealed that εL269F increases the gating equilibrium constant (θ) 30‐fold by increasing β by 8.9‐fold, and decreasing α by 3.3‐fold, and enhancing the agonist affinity (K_d_) by 2.3‐fold (center and right columns in Fig. 7C, and Table 3). The burst duration, predicted by (β/k_–2_ + 1)/α, also agrees with that measured at a low choline concentration (2.80 vs. 2.95 msec) (Tables 2 and 3).

## Discussion

The clinical, electrophysiologic, ultrastructural, and molecular genetics data allowed us to trace the cause of a severe SCCMS to a novel dominant missense mutation, δL237F, in the M2 of the AChR δ subunit. To the best of our knowledge, this is the third report of SCCMS caused by a δ subunit mutation at a novel site.^7^, ^8^, ^9^ In this patient, the safety margin of neuromuscular transmission is compromised by the altered endplate morphology, a depolarization block due to staircase summation of prolonged endplate potentials at physiologic rates of stimulation, and increased desensitization of AChR.

Elucidation of the consequences of SCCMS mutations is challenging because the very brief channel closings are too brief to adequately resolve with present day recording instruments. Among 24 SCCMS mutations reported date, only six were characterized in detail in non‐δ subunits; five of these are in transmembrane domains and one is in the extracellular domain. The αG153S mutation in the extracellular domain enhances agonist binding but does not alter channel gating.^26^ The αN217K mutation in the first transmembrane domain (M1), decreases the agonist dissociation rate.^39^ In M2 domain, the αV249F mutation enhances desensitization and agonist binding affinity,^6^ the εV265A mutation enhances gating efficiency without altering agonist affinity, and the βV266A mutation enhances gating efficiency with only a small increase of agonist affinity.^7^ In the M4 domain, the αC418W mutation enhances gating efficiency without altering agonist binding affinity.^11^ To evaluate which kinetic step or steps are compromised by δL273F, we used the partial agonist choline to generate clusters of channel opening events. This approach revealed that the predominant effect of the mutation is to enhance gating efficiency with only a mild effect on agonist affinity.

The mutant δL273 residue is located near the extracellular end of M2 domain of the δ‐subunit (Fig. 4) and its side chain extends toward the neighboring α‐subunit (Figs. 4D and E). The δL273F mutation markedly increases the channel opening rate and decreases the closing rate. To gain insight into how the mutation affects activation of the receptor, we examined the residues surrounding δL273 using a homology model of the adult human AChR based on high‐resolution crystal structures of related Cys‐loop receptors. In our model, the side chain of δL273 juxtaposes that of αL258 (Fig 4E), suggesting a hydrophobic interaction between the two inter‐helical residues. The δL273F mutation replaces the aliphatic and highly hydrophobic leucine by a larger, aromatic and less hydrophobic phenylalanine.^40^, ^41^ We postulate that the δL273F mutation disrupts the hydrophobic interaction between δL273 and αL258, which destabilizes the closed state and stabilizes the open state of the channel, accounting for the increase in gating efficiency.

The L269F mutation in the M2 domain of ε‐subunit, equivalent to L273F in the δ‐subunit, has been identified in six unrelated SCCMS patients.^9^, ^12^, ^35^, ^36^, ^37^, ^38^ We find that εL269F impairs channel gating less than δL273F, and that synaptic currents in the patient harboring the εL269F mutation are less prolonged than those in the patient harboring the δL273F mutation (Table 1).^12^ In our homology model, the side chain of εL269 juxtaposes that of αL258, establishing a hydrophobic interaction analogous to that between δL273 and αL258 (Fig. 4E), suggesting the inter‐helical hydrophobic interactions between the ε/α and the δ/α subunit pairs are unequal.

## Author contributions

XMS and AGE contributed to study concept and design. XMS, MM, BB, DS, and AGE contributed to acquisition of data. HLW and SMS contributed to structure modeling. All authors contributed to data analysis and interpretation. XMS, SMS, and AGE contributed to drafting and revision of manuscript. XMS, DS, SMS, and AGE contributed to study funding.

## Conflict of interest

AGE receives compensation for serving as Associate Executive Editor for Neuromuscular Disorders. MM receives research support from Mayo Clinic, an MDA care center grant and honorarium to serve as Associate Editor of Neurology Genetics.

## Supporting information


**Data S1**: Supplementary Material
