# Supplementary material for: Slow‐channel myasthenia due to novel mutation in M2 domain of AChR delta subunit
Source: Ann Clin Transl Neurol. 2019 Sep 27;6(10):2066–78. doi: 10.1002/acn3.50902 (PMC6801167; doi:10.1002/acn3.50902)
Supplement: Supplementary file 1 — Data S1: Supplementary Material [file ACN3-6-2066-s001.pdf]

Supplementary Material

APPROVED

Slow-channel myasthenia due to novel mutation in M2 domain of AChR delta subunit

Xin-Ming Shen, PhD[1], Margherita Milone, PhD, MD[1], Hang-Long Wang, PhD[2], Brenda Banwell, MD[3], Duygu Selcen, MD[1], Steven M Sine, PhD[4], and Andrew G Engel, MD[1]

[1]Department of Neurology and Neuromuscular Research Laboratory, Mayo Clinic, Rochester, MN. [2]Department of Neurology and Vesicular Biology Laboratory, Mayo Clinic, Rochester, MN. [3]Division of Neurology, Department of Pediatrics, Children's Hospital of Philadelphia, Philadelphia, PA. [4]Department of Physiology and Biomedical Engineering and Receptor Biology Laboratory; Department of Pharmacology and Experimental Therapeutics; Department of Neurology, Mayo Clinic, Rochester, MN.

Address correspondence to: Dr. Xin-Ming Shen or Dr. Andrew G Engel, Department of Neurology, Mayo Clinic, 200 1st Street SW, Rochester, MN, USA, E-mail: shen.xinming@mayo.edu; age@mayo.edu.

Further description of methods

Structural studies

Intercostal muscle specimens were obtained from the patient and from control subjects without muscle disease undergoing thoracic surgery. Cryosections were used to co-localize the acetylcholine receptor (AChR) and acetylcholine esterase (AChE), as described.[1] EPs were localized for electron microscopy,[2] and quantitatively analyzed by established methods.[3] Peroxidase-labeled α-bgt was used for the ultra-structural localization of AChR.[4] The number of AChRs per EP was measured with $[^{125}I]$α-bgt.[5]

Construction and expression of wild-type and mutant AChR

Sources of human α-, β-, δ-, and ε-subunit cDNAs and subcloning of the cDNAs into the CMV-based mammalian expression vector pRBG4 were as previously described.[6] The mutations were engineered into wild-type AChR subunit cDNAs in pRBG4 using the QuikChange Site-Directed Mutagenesis Kit (Agilent Technologies). Presence of each mutation and absence of unwanted mutations were confirmed by sequencing the entire inserts. HEK293 cells (ATTC)

were transfected with plasmid DNA encoding α-, β-, δ- and ε-subunits, and pEGFP-N1 at a ratio of 2:1:1:1:1, using the FuGENE 6 transfection reagent (Roche).[7]

α-Bungarotoxin binding measurements

The total number of ^{125}I-α-bgt sites on the surface of transfected HEK cells was determined as previously described.[6] Briefly, intact cells were harvested 3 days after transfection by gentle agitation in PBS with 5 mM EDTA. After centrifugation, the cells were resuspended in a high-potassium Ringer's solution and divided into aliquots for measurements of α-bgt binding. The total number of α-bgt binding sites was determined by incubation for 1 hour in the presence of 5 nM ^{125}I-α-bgt. Unbound toxin was removed by washing with high-potassium Ringer's solution containing 300 μM d-tubocurarine, followed by filtration using a cell harvester (Brandel Incorporated). Radioactivity retained by glass fiber filters (Whatman GF-B, 1-μm cutoff) was measured with a gamma counter. Nonspecific binding was determined in the presence of 300 μM d-tubocurarine.

Measurements of ACh competition against ^{125}I-α-bgt binding were analyzed using the Hill equation, $1 - Y = 1/(1 + ([ACh]/K_{ov})^n)$, where Y is fractional occupancy by ACh, n is the Hill coefficient, and K_{OV} is an overall dissociation constant.[8]

Patch-clamp recordings and single-channel kinetic analysis

Recordings were obtained in the cell-attached patch configuration at a membrane potential of -80 mV at 22°C with bath and pipette solutions containing (in mM): 142 KCl, 5.4 NaCl, 1.8 CaCl2, 1.7 MgCl2, 10 HEPES, pH 7.4. Single-channel currents were recorded using an Axopatch 200A amplifier (Axon Instruments) at a bandwidth of 50 kHz, digitized at 5-μs intervals using a Digidata 1322A (Axon Instruments) and recorded to a hard disk using the program Clampex 8.2 (Axon Instruments). Recordings obtained with ACh concentrations of 1 μM or lower were analyzed at a uniform bandwidth of 11.7 kHz with an imposed dead time of 15.3 μs. Recordings obtained with ACh concentrations of 10 μM or greater were analyzed at a bandwidth of 10 kHz and an imposed dead time of 25 μs using TACx4.0.9 software (Bruxton). Dwell-time histograms were plotted on a logarithmic abscissa and fitted with the sum of exponentials by maximum likelihood.[9]

To estimate rate constants underlying AChR activation, we employed desensitizing concentrations of the partial agonist choline.[10-13] High agonist concentrations elicit openings from a single receptor channel that cluster into sequences of identifiable activation episodes.[14] Channel openings elicited by low concentrations of choline (20 to 50 μM) were analyzed at 11.7

kHz. Clusters of channel openings elicited by 0.2 to 20 mM choline were analyzed at 4 kHz due to frequent, brief channel blockages that reduced the single-channel current amplitude. Clusters were identified as a series of closely spaced openings preceded and followed by closed intervals greater than a defined critical time. The critical time was determined by a method that misclassifies an equal number of events between two adjacent closed-time components.[15] For each receptor, the critical time providing the best fit for the closed-time histogram was chosen for the final analysis. Clusters with fewer than 5 openings were excluded from analysis. Individual clusters were examined for homogeneity based on the mean open probability and open duration for each cluster, and clusters within 2 SDs of the mean were accepted for further analysis.[16, 17] The resulting global set of open and closed dwell times from wild-type and mutant AChRs were analyzed using the program MIL (www.qub.buffalo.edu/wiki/index.php/main_page), which uses an interval-based maximum likelihood method that also corrects for missed events to yield fitted rate constants in a kinetic scheme for receptor activation.[16]

References

1. Fambrough, D.M., A.G. Engel & T.L. Rosenberry. 1982. Acetylcholinesterase of human erythrocytes and neuromuscular junctions: homologies revealed by monoclonal antibodies. *Proc. Natl. Acad. Sci. U. S. A*. **79**: 1078-1082.

2. Engel, A.G. 2004. "The muscle biopsy". In *Myology*. A.G. Engel, Franzini-Armstrong, C., Ed.: 681-690. New York, NY: McGraw-Hill.

3. Engel, A.G. 1994. "Quantitative morphological studies of muscle". In *Myology*. A.G. Engel, Franzini-Armstrong C., Ed.: 1018-1045. New York, NY: McGraw-Hill.

4. Engel, A.G., J.M. Lindstrom, E.H. Lambert*, et al.* 1977. Ultrastructural localization of the acetylcholine receptor in myasthenia gravis and in its experimental autoimmune model. *Neurology*. **27**: 307-315.

5. Engel, A.G. 1993. The investigation of congenital myasthenic syndromes. *Ann N. Y. Acad. Sci*. **681**: 425-434.

6. Ohno, K., H.L. Wang, M. Milone*, et al.* 1996. Congenital myasthenic syndrome caused by decreased agonist binding affinity due to a mutation in the acetylcholine receptor epsilon subunit. *Neuron*. **17**: 157-170.

7. Shen, X.M., K. Ohno, S.M. Sine*, et al.* 2005. Subunit-specific contribution to agonist binding and channel gating revealed by inherited mutation in muscle acetylcholine receptor M3-M4 linker. *Brain*. **128**: 345-355.

8. Sine, S.M., P. Quiram, F. Papanikolaou*, et al.* 1994. Conserved tyrosines in the alpha subunit of the nicotinic acetylcholine receptor stabilize quaternary ammonium groups of agonists and curariform antagonists. *J. Biol. Chem*. **269**: 8808-8816.

9. Sigworth, F.J. & S.M. Sine. 1987. Data transformations for improved display and fitting of single-channel dwell time histograms. *Biophys. J*. **52**: 1047-1054.

10. Zhou, M., A.G. Engel & A. Auerbach. 1999. Serum choline activates mutant acetylcholine receptors that cause slow channel congenital myasthenic syndromes. *Proc. Natl. Acad. Sci. U. S. A*. **96**: 10466-10471.

11. Grosman, C. & A. Auerbach. 2000. Asymmetric and independent contribution of the second transmembrane segment 12' residues to diliganded gating of acetylcholine receptor channels: a single-channel study with choline as the agonist. *J. Gen. Physiol*. **115**: 637-651.

12. Shen, X.M., F. Deymeer, S.M. Sine*, et al.* 2006. Slow-channel mutation in acetylcholine receptor alphaM4 domain and its efficient knockdown. *Ann Neurol*. **60**: 128-136.

13. Shen, X.M., T. Okuno, M. Milone*, et al.* 2016. Mutations Causing Slow-Channel Myasthenia Reveal That a Valine Ring in the Channel Pore of Muscle AChR is Optimized for Stabilizing Channel Gating. *Hum Mutat*. **37**: 1051-1059.

14. Sakmann, B., J. Patlak & E. Neher. 1980. Single acetylcholine-activated channels show burst-kinetics in presence of desensitizing concentrations of agonist. *Nature*. **286**: 71-73.

15. Colquhoun, D. & B. Sakmann. 1985. Fast events in single-channel currents activated by acetylcholine and its analogues at the frog muscle end-plate. *J. Physiol*. **369**: 501-557.

16. Qin, F., A. Auerbach & F. Sachs. 1997. Maximum likelihood estimation of aggregated Markov processes. *Proc. Biol. Sci*. **264**: 375-383.

17. Shen, X.M., K. Ohno, T. Fukudome*, et al.* 2002. Congenital myasthenic syndrome caused by low-expressor fast-channel AChR delta subunit mutation. *Neurology*. **59**: 1881-1888.
